# Functional Coupling between the Fronto-Parietal Network and Default Mode Network Is Associated with Balanced Time Perspective

**DOI:** 10.3390/brainsci12091201

**Published:** 2022-09-06

**Authors:** Tao Chen, Jia Huang, Ji-fang Cui, Zhi Li, Ya Wang, Muireann Irish, Raymond C. K. Chan

**Affiliations:** 1 Neuropsychology and Applied Cognitive Neuroscience Laboratory, CAS Key Laboratory of Mental Health, Institute of Psychology, Beijing 100101, China; 2 Department of Psychology, University of Chinese Academy of Sciences, Beijing 101408, China; 3 Brain and Mind Centre, The University of Sydney, Sydney 2006, Australia; 4 School of Psychology, The University of Sydney, Sydney 2006, Australia; 5 Institute of Educational Information and Statistics, National Institute of Education Sciences, Beijing 100098, China

**Keywords:** fronto-parietal network, default mode network, balanced time perspective, cognitive control

## Abstract

Balanced time perspective refers to the ability to flexibly switch between different temporal foci in an adaptive manner according to the current context. Functional connectivity within the default mode network (DMN) has been suggested to support balanced time perspective. The coupling between the DMN and fronto-parietal network (FPN) may drive many important expressions of internally directed cognition. However, it remains unclear whether balanced time perspective is supported by the interaction between the FPN and DMN. To examine these issues, we recruited 91 participants (52 males with mean age of 19.6, and 39 females with mean age of 20.0) to undergo resting-state brain imaging scan and to complete a questionnaire measuring balanced time perspective. Seed-based voxel-wise functional connectivity analyses implicated midline DMN regions including the anterior medial prefrontal cortex (amPFC) and posterior cingulate cortex (PCC) along with the anterior cingulate cortex (ACC), precuneus, and cerebellum in supporting a balanced time perspective. More importantly, functional connectivity between the right amPFC and right dorsal lateral prefrontal cortex (DLPFC) in the FPN was found to associate with balanced time perspective. Our findings suggest the importance of coordinated brain activity in supporting a balanced time perspective.

## 1. Introduction

Time perspective (TP) is defined as an individual’s relative investment of attention on different temporal contexts (e.g., past, present and future) and is posited to influence goal-directed behaviour and wellbeing [1]. Zimbardo and Boyd [2] proposed five dimensions of TP: Future (a fixation on future goals at the expense of present enjoyment); Past-Negative (a negative view of the past); Past-Positive (a positive attitude towards the past); Present-Fatalistic (a hopeless and helpless view to life) and Present-Hedonistic (indulging in the present while ignoring negative consequences). Typically, individuals with more Past-Positive, Present-Hedonistic and Future TP display better mental health and report higher life satisfaction [3], suggesting an important relationship between TP and functional outcomes in healthy individuals.

Importantly, however, the ability to hold multiple temporal contexts in mind and to flexibly shift our thoughts and behaviours in the service of current and future goals has been posited as the most adaptive expression of TP [1]. Proposed as a key component of positive psychology [1,4], balanced TP is associated with a range of domains relevant for wellbeing including life satisfaction [5,6,7,8]. Recent studies suggest that balanced TP is associated with better mental health (e.g., reduced anxiety and depression symptoms) [9,10,11] and psychological flexibility [12]. In addition, participants with more balanced TP have been shown to display greater self-control [13], higher levels of emotional intelligence [14] and mindfulness [15]. By the same token, deviations from balanced TP are suggested to relate to poorer mental state. Traffic accident survivors with more serious posttraumatic stress disorder (PTSD) symptoms have been shown to report less balanced TP [16] as do individuals with schizophrenia [17]. As such, the literature converges to suggest the importance of maintaining several temporal perspectives in mind at any given time to flexibly shift between these perspectives depending on current goals [1].

Despite its importance for a range of adaptive outcomes, surprisingly little is known about the underlying neural mechanisms of balanced TP. This is particularly the case from a brain network perspective, with a paucity of studies exploring the interplay between large-scale brain networks in supporting balanced TP. Preliminary studies suggest that the default mode network (DMN) plays a crucial role in supporting balanced TP, whereby stronger associations between the precuneus and other regions of DMN (e.g., bilateral temporoparietal junction) are associated with more balanced TP [9,18].

The DMN is of particular interest in this context as it is widely held to support complex expressions of internally driven cognition [19]. The DMN can be parcelled into three functionally distinct subsystems: dorsal medial prefrontal cortex (dmPFC) subsystem; a medial temporal lobe (MTL) subsystem; and a midline core comprising the posterior cingulate cortex (PCC) and anterior medial prefrontal cortex (amPFC) [20]. Specifically, these midline regions are proposed to play a central role as hubs connecting the other two subsystems [20] to support a range of complex cognitive capacities including autobiographical memory, future thinking, theory of mind, and mind wandering [21,22,23]. Given these diverse introspective functions, which are often focused on a particular temporal context, it is not surprising that previous studies have reported significant DMN involvement associated with a balanced TP [9,18].

In addition to the DMN, accumulating evidence also points to a fundamental contribution of the frontoparietal control network (FPN), the key substrate of cognitive function [24], in supporting internally directed cognition, especially intentional or goal-directed types [25,26,27]. Empirical findings also suggest that the coupling between DMN and FPN may drive many important expressions of internally directed cognition, such as mind wandering [28]. To strike a balance between inhibiting habitual and maladaptive TPs versus representing and switching to the most appropriate TP for a given context, it seems intuitive to propose that executive control would be required.

Christoff., et al. [27] proposed that when individuals allocate cognitive resources to support internally directed cognition, FPN would couple with the core of DMN (including PCC, amPFC). The current study therefore aimed to use PCC and amPFC as the seeds of DMN to examine the resting-state functional connectivity (rsFC) between FPN and DMN underlying balanced TP.

## 2. Methods

### 2.1. Participants

A total of 105 participants underwent TP evaluation, described in full elsewhere [29]. Ninety-one participants were involved in the final rsFC analysis as 14 participants were removed due to large head movement (exclusion criteria: head-motion > 2 mm or rotation > 2°) during the MRI scan or poor imaging quality. The remaining 91 participants, including 52 males and 39 females, had a mean age of 19.8 years (SD = 2.12, ranging from 16–26 years). The average length of education was 13.0 years (SD = 1.92) and ranged from 9 to 19 years. Participants were excluded if they reported a history of psychiatric/neurological disorders or a family history of psychiatric disorders, drug or alcohol abuse/dependence. The study was performed following the Declaration of Helsinki and was approved by the ethics committee of the Institute of Psychology, Chinese Academy of Sciences (H15043). Participants provided written informed consent before the study began.

### 2.2. Cognitive Measures

Time perspective was assessed by the 20-item Chinese brief version of the Zimbardo Time Perspective Inventory (ZTPI), which shows good reliability and validity in the Chinese context [2,30]. The Chinese version of ZTPI is composed of five distinctive dimensions: Past-Positive, Past-Negative, Future, Present-Hedonistic and Present-Fatalistic. For example, an item from the Past-Positive dimension is “Happy memories of good times spring readily to mind.” It requires participants to respond to each item on a 5-point scale (1 = Very uncharacteristic of me, 5 = Very characteristic of me).

Deviation from the Balanced Time Perspective (DBTP) indicates how far the empirical TP profile approximates the optimal TP profile [31]. The formula for calculating DBTP is:

$$DBTP=\sqrt{{\left(oPN-ePN\right)}^{2}+{\left(oPP-ePP\right)}^{2}+{\left(oPF-ePF\right)}^{2}+{\left(oPH-ePH\right)}^{2}+{\left(oF-eF\right)}^{2}}$$

Specifically, *oPN* (1.95), *oPP* (4.60), *oPF* (1.50), *oPH* (3.90), *oF* (4.00) represent optimal scores of Past-Negative, Past-Positive, Present-Fatalistic, Present-Hedonistic, and Future TP, respectively, while *ePN*, *ePP*, *ePF*, *ePH*, *eF* represent scores of Past-Negative, Past-Positive, Present-Fatalistic, Present-Hedonistic, and Future TP reported by participants [31]. DBTP, calculated from the ZTPI, is viewed as the optimal indicator of balanced TP [32], and has been widely used in Chinese populations [5,9,18,33]. A lower DBTP score indicates a more balanced TP.

### 2.3. Brain Imaging Data Acquisition

MR imaging data were acquired with a Siemens 3T scanner (SIEMENS 3T-Trio A Tim, Erlangen, Germany) using a 32-channel head coil at 306 Hospital, Beijing. Magnetization Prepared Rapid Acquisition Gradient-Echo (MPRAGE) sequence (TR: 2300 ms, TE: 3.01 ms, flip angle: 9°, FOV: 240 × 256 mm, matrix = 256 × 256, voxel size: 1 × 1 × 1 mm^3^) was used to obtain high-resolution T1-weighted anatomical images. T2-weighted echo planar imaging (EPI) sequence (TR: 2000 ms, TE: 30 ms, flip angle: 90°, resolution matrix = 64 × 64, FOV: 210 × 210 mm, 32 slices, voxel size: 3.3 mm × 3.3 mm × 4 mm) was used to collect resting-state images. The resting-state scan lasted for approximately 6 min and included 180 volumes. Participants were asked to stay awake with eyes closed during the resting-state imaging scan.

### 2.4. Regions of Interest (ROI)

**Figure 1:**
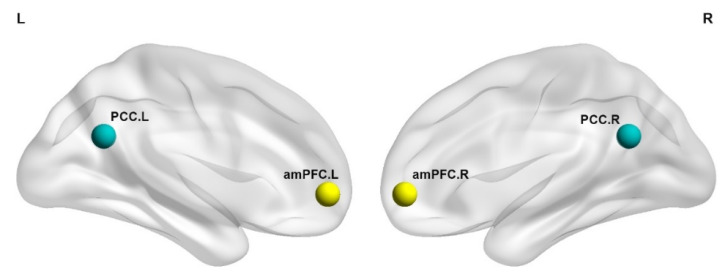
Schematic of ROIs in DMN core regions used in the current study. amPFC = anteromedial prefrontal cortex; PCC = posterior cingulate cortex; L = left; R = right.

ROI included DMN midline core regions: PCC, amPFC in both hemispheres. Spherical ROIs (4 mm diameter) of the DMN core were created with the coordinates reported by Andrews-Hanna, Reidler [20] for amPFC (left: -6 52 -2; right: 6 52 -2) and PCC (left: -8 -56 26; right: 8 -56 26), as presented in Figure 1.

### 2.5. Brain Imaging Data Pre-Processing

Resting-state brain images were pre-processed using the Statistical Parametric Mapping Software (SPM12, http://www.fil.ion.ucl.ac.uk/spm, accessed on 5 February 2021) and Data Processing & Analysis for Brain Imaging Software (DPABI 5.1, http://www.rfmri.org/dpabi, accessed on 5 February 2021) [34] running in MatLab R2013b (The MathWorks, Inc., Natick, MA, USA).

Pre-processing steps included: (1) discarding the first 10 volumes; (2) slice timing; (3) head motion correction with an exclusion criteria: head-motion > 2 mm or rotation > 2°; (4) co-registering the T1-structural images to the functional images and segmenting T1-structural images into gray matter (GM), white matter (WM) and cerebral spinal fluid (CSF); (5) normalizing to the MNI template using segmented information; (6) resampling into 3 mm × 3 mm × 3 mm resolution; (7) smoothing with a 4-mm full-width at half maximum isotropic Gaussian kernel; (8) eliminating linear trends; (9) nuisance covariate regression (including Friston’s 24-parameter model, the white matter signal, global mean signal and cerebrospinal fluid signal); and (10) temporal filtering (0.01–0.1 Hz).

### 2.6. Voxel-Wise Resting-State Functional Connectivity (rsFC) Analysis

SPM12, DPABI 5.1 [34] running in MatLab R2013b were used to perform the seed based voxel-wise rsFC analysis. Four spherical ROIs (4 mm diameter) including amPFC (left: -6 52 -2; right: 6 52 -2) and PCC (left: -8 -56 26; right: 8 -56 26) were set as seeds. The rsFC maps were generated by computing the correlation between the average time series of each seed and the whole brain voxels, the correlation coefficients were then transformed to normally distributed Z-scores via Fisher-Z transform approach. Finally, rsFC significantly correlating with DBTP, was identified by multiple regression analysis. Thresholds of voxel-level *p* < 0.001 uncorrected and cluster-level *p* < 0.05 with FWE correction were applied for multiple comparisons, in keeping with previous studies.

## 3. Results

### 3.1. Descriptive Information of DBTP

DBTP was normally distributed (Shapiro–Wilk W = 0.985, *p* = 0.373). The average DBTP was 2.10 (SD = 0.76) with a range from 0.37 to 3.84. The skewness and kurtosis were −0.16 and −0.60, respectively.

### 3.2. Correlations between Voxel-Wise rsFC and DBTP

**Figure 2:**
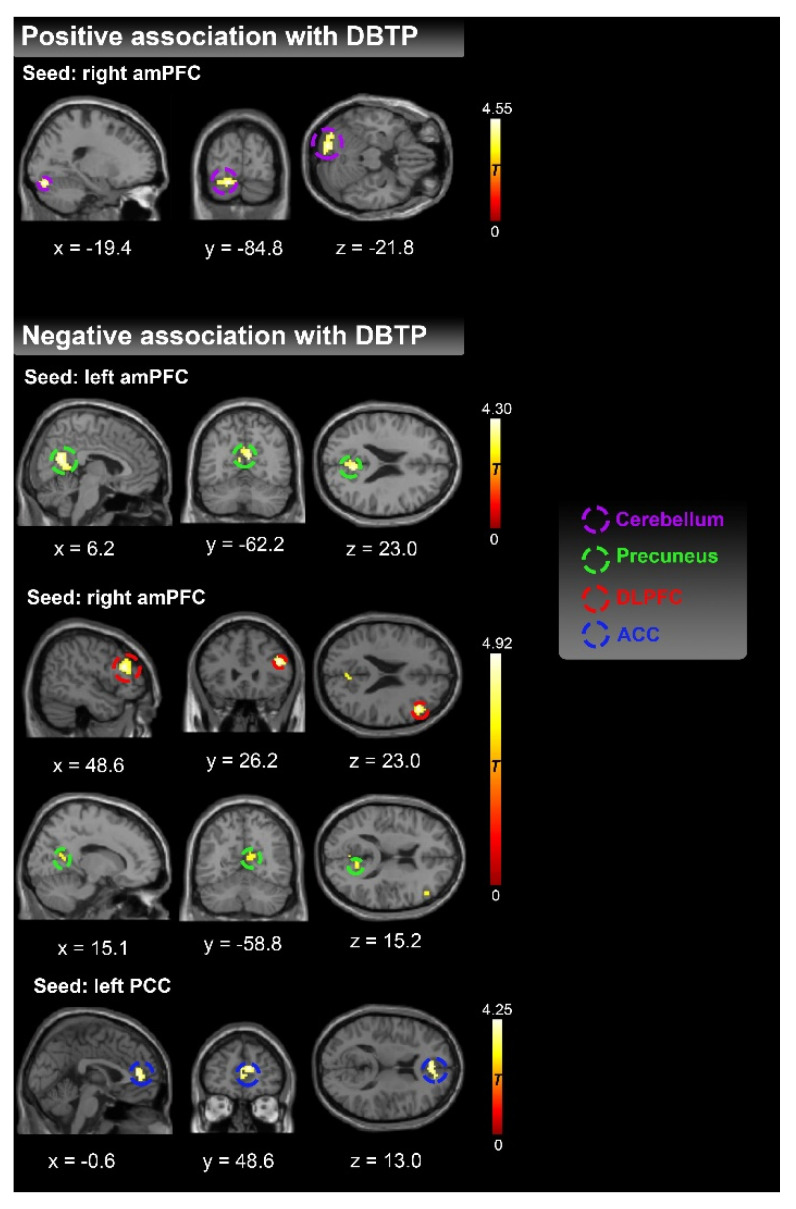
Resting state functional connectivity involving DMN core seeds (PCC, amPFC in both hemispheres) showing significant correlations with DBTP. DMN = default mode network, amPFC = anterior medial prefrontal cortex, PCC = posterior cingulate cortex, DLPFC = dorsal lateral prefrontal cortex, ACC = anterior cingulate cortex. DBTP = Deviation from Balanced Time Perspective.

**Figure 3:**
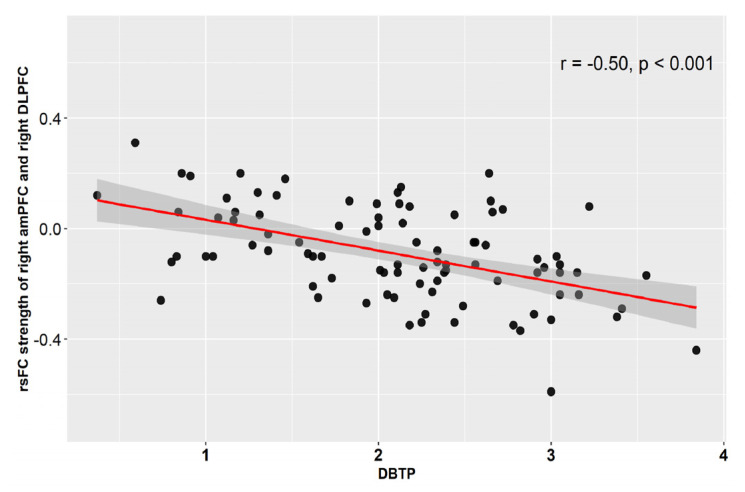
Resting state functional connectivity strength of nodes in DMN (i.e., right amPFC) and FPN (i.e., right DLPFC) showing significant negative correlation with DBTP. DMN = default mode network, FPN = frontoparietal control network, rsFC = resting state functional connectivity (Z-score), amPFC = anterior medial prefrontal cortex, DLPFC = dorsal lateral prefrontal cortex. DBTP = Deviation from Balanced Time Perspective.

As presented in Table 1 and Figure 2, DBTP was negatively correlated with rsFC between left amPFC and bilateral precuneus. We also found DBTP negatively correlated with the rsFC between right amPFC and right precuneus, while the DBTP was positively correlated with the rsFC between right amPFC and left cerebellum exterior. In addition, DBTP was found to negatively correlate with the rsFC between left PCC and bilateral anterior cingulate gyrus. The scatter plot for the above results is presented in Figure S1 in the Supplementary Material. Moreover, a significant negative correlation between DBTP and right amPFC-right DLPFC connectivity was observed, and the scatter plot is displayed in Figure 3. No other associations emerged as significant. The results remain stable after controlling for age and education years.

## 4. Discussion

The aim of this study was to examine the involvement of established large-scale brain networks, including the DMN and the FPN, in supporting a balanced time perspective (TP). Overall, rsFC analyses suggest that the coupling of key regions within the FPN and DMN supports the maintenance of a balanced TP.

The DMN has received widespread attention for its role in mediating various forms of internally directed cognition [22,28,35,36]. Notably, the amPFC and PCC, as the putative midline core of the DMN [19], have been proposed to subtend self-referential forms of processing, as well as coordinating activity across the DMN more broadly, while the precuneus is posited to support visuospatial processing and mental imagery [37,38]. It stands to reason that the DMN should be related to the efficiency of maintaining a balanced TP given the requirement for introspection, prospection, and maintenance of self-referential forms of imagery [21,37]. Indeed, previous studies adopting rsFC analysis revealed that connectivity between PCC with parahippocampal gyrus, and connectivity between precuneus and mPFC correlated with balanced TP [9,18]. Here we demonstrated that functional connectivity between DMN core regions (including amPFC, PCC) as well as other key structures within DMN putative subsystems were implicated in maintaining a balanced TP.

Considering first the amPFC, we found that stronger connectivity between left amPFC and bilateral precuneus was associated with a more balanced TP, resonating with previous studies [18]. We further found evidence for functional connectivity between the PCC and ACC in supporting a balanced TP. The PCC is one of the major hubs of the DMN and contributes to internally directed cognition [39], while the rostral ACC may facilitate adaptive interpretation of negative feelings and anticipatory planning during self-reflection [40]. Thus, the increasing rsFC between left PCC and bilateral ACC may support regulation of negative emotions during internal representation to engage in adaptive forms of mentation. Interestingly, empirical evidence has related aspects of emotion regulation to balanced TP [14] offering some preliminary support for this proposal.

An unexpected finding was that DBTP was positively correlated with the functional connectivity between right amPFC and left cerebellum. Increased cerebellar–medial prefrontal cortex functional connectivity has been observed in several clinical disorders including major depressive disorder [41,42] and has been interpreted as a functional compensation [42]. It is increasingly recognised that individuals with less balanced TP are more inclined to suffer from depressive symptoms [5,43], suggesting a potential link between these two findings. Future studies, however, will be required to formally reconcile these speculations. In addition, the cluster extended from cerebellum to occipital fusiform gyrus and lingual gyrus, therefore, we need to interpret the findings with caution.

It is now well-established that, alongside the DMN, the FPN plays an active role in supporting intentional internally directed forms of cognition [25,26,27]. In the present study, we found that DBTP negatively correlated with functional connectivity between the right amPFC and right DLPFC. The DLPFC is the hub of the FPN control network [24] and modulates executive control [44]. To ensure the optimal TP according to the current context, habitual TPs must be inhibited, while the appropriate TP is represented. Then one must flexibly switch to, and adopt, the preferred TP, thus drawing upon a diverse set of cognitive processes which require a high level of cognitive control [14,45,46]. Our findings of increased functional coupling between the amPFC and right DLPFC in relation to balanced TP thus resonates with the existing literature on cognitive control; however, it will be important to formally test the role of cognitive control in relation to FPN-DMN coupling and TP using targeted tasks.

Our findings have a number of theoretical and clinical implications. First, the results deepen our understanding of the interplay between DMN and FPN by delineating the interplay between FPN and DMN in supporting a balanced TP. Balanced TP is linked with well-being and a range of adaptive outcomes [5,32], and represents an important aspect of internal mentation that warrants further empirical study. The current findings may enable us to understand the neurobiology of deviations from a balanced TP, particularly in relation to clinical disorders with alterations in FPN and DMN functional connectivity [47]. In the long-term, it may be possible to develop tailored interventions to support a more balanced TP.

A number of methodological limitations warrant consideration, not least our relatively small sample size. Future studies incorporating larger samples spanning a broad range of ages and levels of executive control would provide valuable insights regarding the relationship between functional connectivity, cognitive control, and balanced TP.

In addition, the self-reported checklist of balanced TP is vulnerable to subjective bias. As such, there is a need to develop valid performance-based tasks to objectively measure balanced TP. Furthermore, given that the present study was limited to a non-clinical sample, the association between balanced TP and functional connectivity found in the present study may not generalize to clinical populations. This is particularly the case for clinical disorders showing prospection and mental time travelling impairments, such as dementia [48,49] in which executive control impairments are also observed.

## 5. Conclusions

Our study reveals a significant negative correlation between DBTP and right amPFC-right DLPFC connectivity based on resting-state fMRI, demonstrating the interplay between the DMN and FPN networks in supporting a balanced TP in healthy individuals. Our findings offer new insights into the neurocognitive architecture of a balanced TP as well as potential avenues for intervening when this capacity goes awry.

## Figures and Tables

**Table 1:** Resting state functional connectivity involving DMN core seeds showing significant correlations with DBTP.

Representative Region of the Cluster	Co-Ordinates of Maximum Voxel	Cluster Size (Voxels)	Anatomical Region	*T*/Z Value
**Positive association with DBTP**				
**Seed: right amPFC**				
Left Cerebellum Exterior	−18; −81; −21	82	Left Cerebellum Exterior (41%), Left occipital fusiform gyrus (38%), Left lingual gyrus (16%)	4.55/4.30
**Negative association with DBTP**				
**Seed: left amPFC**				
Bilateral precuneus	9; −57; 15	140	Right precuneus (41%), left precuneus (24%), Right Cuneus (10%)	4.30/4.09
**Seed: right amPFC**				
Right DLPFC	48; 30; 21	73	Right middle frontal gyrus (74%)	4.92/4.61
Right precuneus	12; −57; 15	51	Right precuneus (61%), Right Cuneus (12%)	3.90/3.74
**Seed: left PCC**				
Bilateral ACC	15; 42; 18	77	Right anterior cingulate gyrus (27%), Right superior frontal gyrus medial segment (25%), Left anterior cingulate gyrus (21%)	4.25/4.04

Note: amPFC = anterior medial prefrontal cortex, PCC = posterior cingulate cortex, DLPFC = dorsal lateral prefrontal cortex, ACC = anterior cingulate cortex. DBTP = Deviation from the Balanced Time Perspective.

## Data Availability

Data sharing not applicable.

## Supplementary Materials

The following supporting information can be downloaded at: https://www.mdpi.com/article/10.3390/brainsci12091201/s1, Figure S1: Resting state functional connectivity involving DMN core seeds (PCC, amPFC in both hemispheres) showing significant correlations with DBTP.
