# Supplementary material for: Functional Coupling between the Fronto-Parietal Network and Default Mode Network Is Associated with Balanced Time Perspective
Source: Brain Sci. 2022 Sep 6;12(9):1201. doi: 10.3390/brainsci12091201 (PMC9496926; doi:10.3390/brainsci12091201)
Supplement: Supplementary file 1 [file brainsci-12-01201-s001.zip › brainsci-1884521-supplementary.pdf]

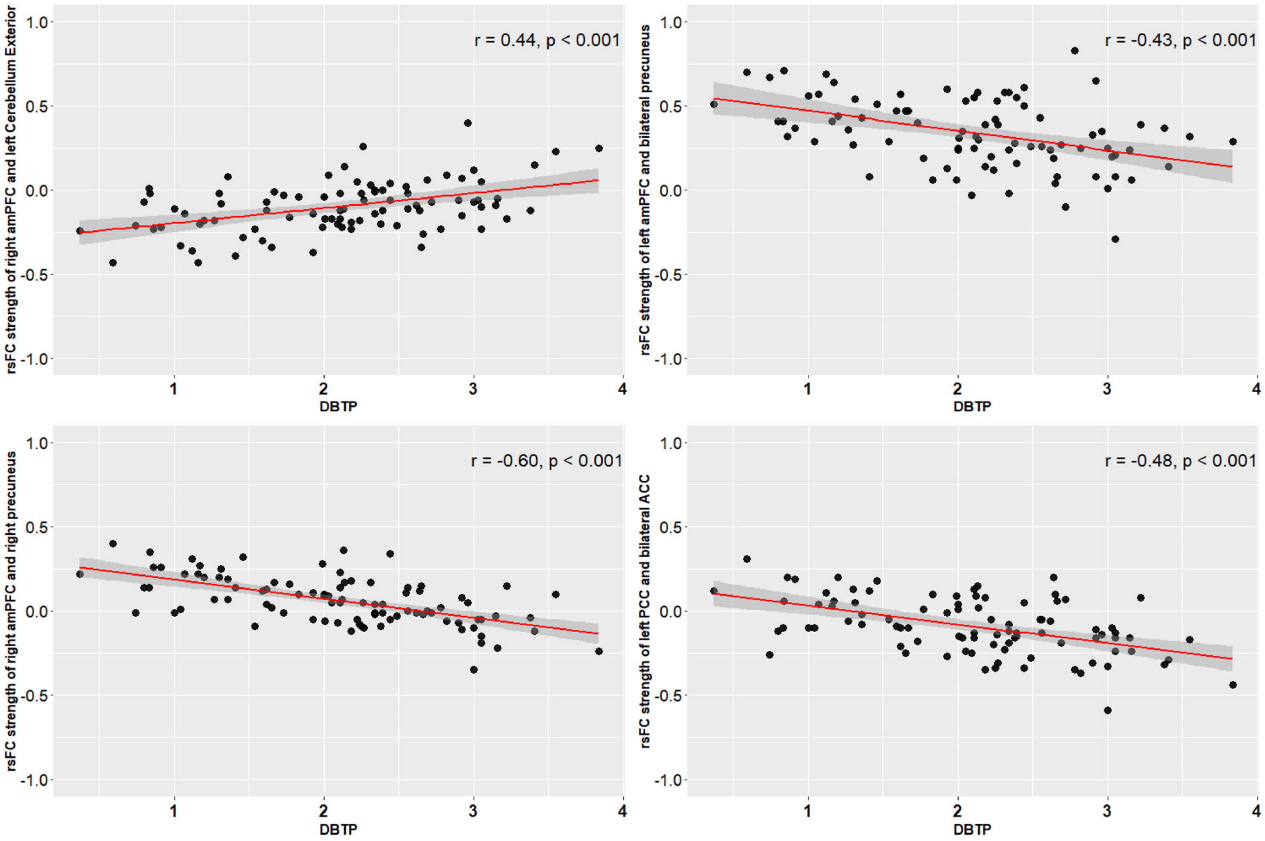

Figure S1. Resting state functional connectivity involving DMN core seeds (PCC, amPFC in both hemispheres) showing significant correlations with DBTP. rsFC = resting state functional connectivity (Z-score). DMN = default mode network, amPFC = anterior medial prefrontal cortex, PCC = posterior cingulate cortex, DLPFC = dorsal lateral prefrontal cortex, ACC = anterior cingulate cortex. DBTP = Deviation from Balanced Time Perspective.
